# Ecosystem services in vineyard landscapes: a focus on aboveground carbon storage and accumulation

**DOI:** 10.1186/s13021-020-00158-z

**Published:** 2020-11-03

**Authors:** J. N. Williams, J. A. Morandé, M. G. Vaghti, J Medellín-Azuara, J. H. Viers

**Affiliations:** 1 Pacific Agroecology LLC, Davis, CA 95616 USA; 2 Department of Environmental Science and Policy, University of California, Davis, CA USA; 3 Department of Civil and Environmental Engineering, University of California, Merced, USA

**Keywords:** Biodynamic farming, Carbon storage rate, Climate mitigation, Grape vine, Organic farming, Regenerative agriculture, Vegetation buffer, Wildland conservation, Woody biomass

## Abstract

**Background:**

Organic viticulture can generate a range of ecosystem services including supporting biodiversity, reducing the use of conventional pesticides and fertilizers, and mitigating greenhouse gas emissions through long-term carbon (C) storage. Here we focused on aboveground C storage rates and accumulation using a one-year increment analysis applied across different winegrape varietals and different-aged vineyard blocks. This produced a chronosequence of C storage rates over what is roughly the productive lifespan of most vines (aged 2–30 years). To our knowledge, this study provides the first estimate of C storage rates in the woody biomass of vines. Additionally, we assessed C storage in wildland buffers and adjacent oak-dominated habitats over a 9-year period.

**Results:**

Carbon storage averaged 6.5 Mg/Ha in vines. We found the average annual increase in woody C storage was 43% by mass. Variation correlated most strongly with vine age, where the younger the vine, the greater the relative increase in annual C. Decreases in C increment rates with vine age were more than offset by the greater overall biomass of older vines, such that C on the landscape continued to increase over the life of the vines at 18.5% per year on average. Varietal did not significantly affect storage rates or total C stored. Carbon storage averaged 81.7 Mg/Ha in native perennial buffer vegetation; we found an 11% increase in mass over 9 years for oak woodlands and savannas.

**Conclusions:**

Despite a decrease in the annual rate of C accumulation as vines age, we found a net increase in aboveground C in the woody biomass of vines. The results indicate the positive role that older vines play in on-farm (vineyard) C and overall aboveground accumulation rates. Additionally, we found that the conservation of native perennial vegetation as vineyard buffers and edge habitats contributes substantially to overall C stores. We recommend that future research consider longer time horizons for increment analysis, as this should improve the precision of C accumulation rate estimates, including in belowground (i.e., soil) reservoirs.

## Background

Agriculture plays a major role in the storage, release and cycling of greenhouse gases [1–3]. From the emission of powerful heat-trapping gases like methane and nitrogen oxides to the storage and sequestration of atmospheric carbon in woody plant tissue and soils, agricultural production in general, and certain crops in particular, have the potential to exacerbate or mitigate greenhouse gas levels [4–6]. Among the crops with promise for mitigation are those that store carbon over multi-year time horizons in woody tissue, including perennial tree, shrub and vine crops such as fruit and nut orchard species, plantation forestry species, and wine and table grapes [7–9].

In Mediterranean-type climates, vineyard cropping systems can make up a significant fraction of agricultural lands. In California, for example, table and wine grapes are the second leading agricultural commodity in sales after dairy, occupying 359,360 hectares (880,000 acres) of cultivated land [10, 11]. To harness the mitigation potential of viticulture, it is necessary to improve our understanding of the specifics of carbon storage in vineyards, including the reservoirs where it occurs, the capacity of those reservoirs, rates of accumulation, and any management practices or other factors that affect those rates [12, 13].

Vineyards are proven long-term bellwethers of environmental conditions [14]. With the multi-decade, and even multi-century, longevity of grapevines and reported positive correlations between vine age, productivity and quality for at least some varietals [15–17], there is practical value in being able to accurately estimate carbon storage rates in long-term reservoirs, such as the woody biomass of vines and vineyard soils. Additionally, vineyards—especially those in established wine growing regions—may represent long-term, multi-generational stable land use types where carbon accumulates to significant levels (i.e., comparable to or greater than the amount in vine blocks) in the surrounding non-vine vegetation and soils for decades or centuries [18]. Previous studies have provided estimates of the carbon stored in working vineyards through snap-shot measurements in vines and soils, and have looked at how that compares to surrounding natural vegetation [18, 19]. Additionally, Brunori et al. [12] estimated whole-plant carbon storage rates for vines in central Italy using carbon fixation models and destructive sampling. To our knowledge, however, no study has yet estimated carbon storage rates in vines based on direct growth increment measurements, nor have researchers explicitly examined how carbon storage varies according to vine age and varietal.

While the woody biomass of vines represents a smaller carbon reservoir in the vineyard landscape compared to that of soil [18], it is more concentrated, easier to measure, and perhaps more intuitive to think about in terms of how it fluctuates with time, management, and factors such as climate and pathogens. Studies at the vineyard scale [12] and at the individual vine scale [19] have provided breakdowns of the relative contribution of the above- and below-ground components of grape vines, and as a result the potential of different management actions (e.g., pruning, head training) to affect woody biomass. It has yet to be shown, however, how vine woody biomass accumulates carbon over time, and the extent to which factors such as management type, varietal and vine age affect this process.

This study makes progress in that direction by providing the first field-based estimates of how annual carbon increment varies in woody biomass for grape vines of different ages and varietals. We present results from a northern California organic vineyard with hopes that similar studies emerge in other vineyard and perennial woody crop landscapes across a range of management types (e.g., organic, conventional) to give greater context to these findings. Together, such studies will improve our understanding of how geography, environment and management can be harnessed to advance the much-needed goal of expanding the carbon mitigation potential of agricultural landscapes [20, 21] and augment the generation of ecosystem services [22].

## Methods

### Site description

Vineyard lands sampled in this study are located in the Russian River watershed near the town of Hopland (38°58′23″N 123°06′59″W) in Mendocino County, California. The region is characterized by a Mediterranean-type climate with cool, wet winters and hot, dry summers where the average monthly temperature high/low for the coldest month (December) is 14°/8° C, and for the warmest month (July) is 35°/21° C. Annual rainfall is approximately 1010 mm, with most of that falling between the months of December and March. Vineyard elevations vary from a low of 150 m asl at the valley bottom to roughly 800 m asl at the highest site.

In the study region we sampled vines and wildlands from 12 distinct properties, or ranches, where each ranch exclusively used one the following management regimes: organic (n = 9); and biodynamic (also organic compliant) (n = 3). The ranches are further divided into vineyard blocks—each of which is a consolidated area of ≤ 10 hectares with similar conditions (slope, aspect, soil type), generally planted in a single varietal of vines of the same age. The ranches under organic management follow farming protocols set by the National Organic Program of the United States Department of Agriculture (USDA) and certified by California Certified Organic Farmers (www.ccof.org), while the ranches under biodynamic management (a form of organic management with additional requirements; see https://www.biodynamics.com/biodynamic-principles-and-practices) follow protocols set and certified by Demeter (www.demeter-usa.org). Wildland buffers were classified into five vegetation types [23, 24]: Russian River riparian; creek riparian; oak savannah; oak woodland; and oak madrone woodland.

## Sampling approach

### Vine woody carbon & increment

Biometric measurements of vines were taken in Year-0 and Year-1 for 464 grapevines. These measurements were used in conjunction with allometric relationships and experimentally derived estimates of vine density to calculate above- and below-ground woody biomass and C content. The vines were planted between 1987 and 2015, representing 16 different varietals and managed using one of the above-mentioned management regimes.

To quantify the annual C accumulation and increment on a vineyard block, we first established baseline quantities of long-term (slowly decomposing) C storage in the woody biomass of vines. These initial estimates of C were obtained for each block, characterized according to winegrape varietal and vine age. The data collected were entered into a geographic information system (GIS) for subsequent extrapolation across the landscape with a multivariable spatial model. Vine woody carbon was calculated as the sum of the aboveground trunk and cordons plus the belowground stump and root ball as detailed below.

#### Aboveground

**Fig. 1:**
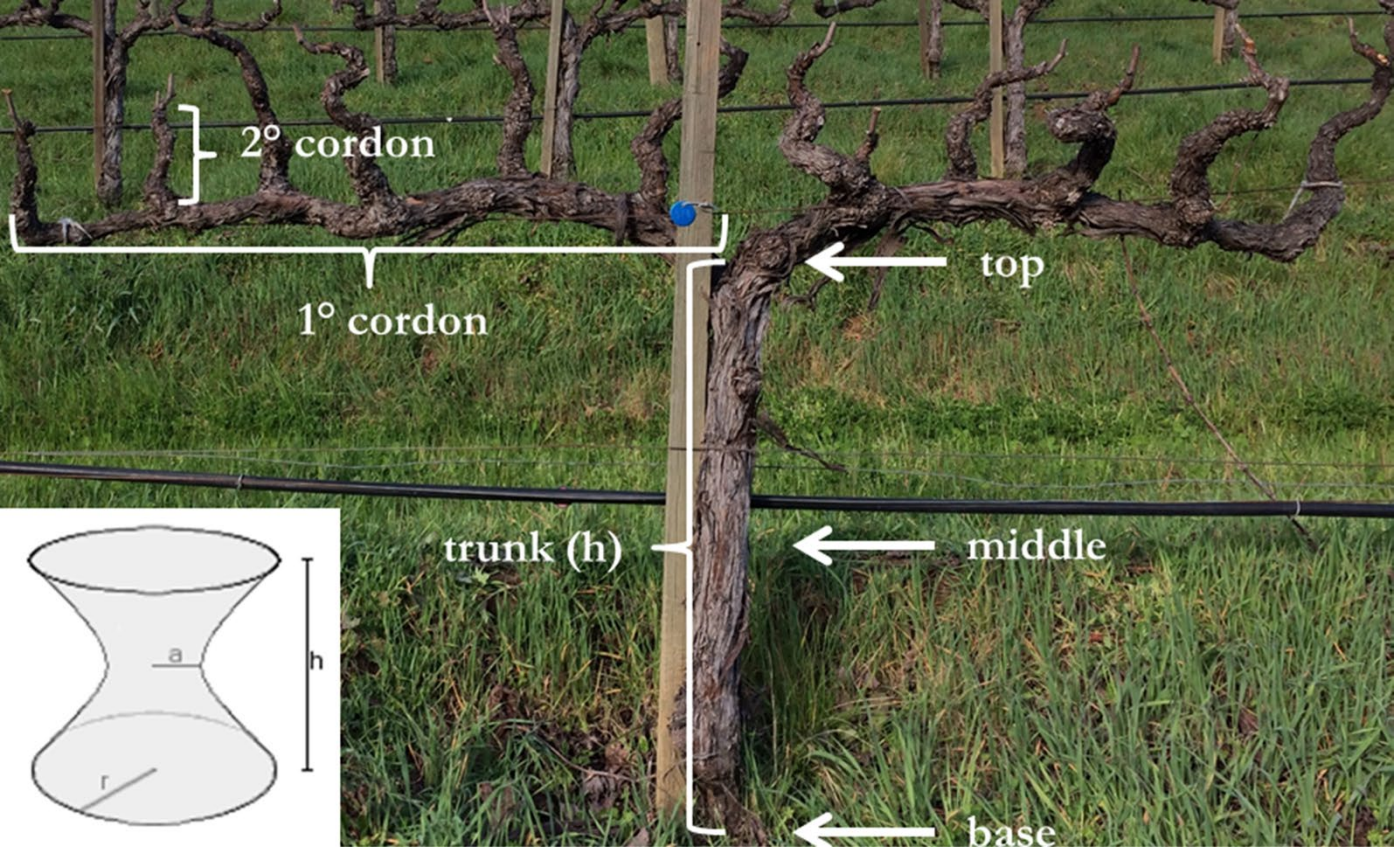
Aboveground vine measurement protocol. This grape vine shows where length (brackets) and diameter (arrows) measurements were taken to calculate perennial aboveground wood volume. The thin branches above the secondary cordon are remnants of annual canes which are not included in the estimate. The inset shows single sheet hyperboloid used to estimate main trunk volume. The round blue tag has unique identification number for subsequent measurements

We estimated aboveground carbon in woody biomass of grapevines where, for each vine measured, three vine trunk diameter measurements were taken: one above the graft node; one just below the cordon split; and one at a point intermediate between those two points (Fig. 1). Additionally, primary and secondary cordon lengths and diameters were measured. All vine-related diameters were measured using calipers, while vine height and cordon lengths were measured using a standard flexible measuring tape. At Year-0, we attached a metal tag with a unique number to each vine to facilitate identification and remeasurement at Year-1.

Per-vine wood volume was estimated as the sum of the volumes of the main trunk plus the perennial cordons and sub-cordons, but not the annual canes (Fig. 1). For the main trunk, volume was estimated using the formula of a single sheet hyperboloid:

$${Volume}_{Hyperboloid}= \frac{\pi *h}{3}*\left(2{{r}_{m}}^{2}+ {{r}_{b}}^{2}\right)$$

where height (*h*) is the length of the main trunk, the middle radius (*r*_*m*_) is taken at the trunk mid-point (*a* in the inset of Fig. 1) and the basal radius (*r*_*b*_) is taken near the ground just above the graft node (*r* in the inset of Fig. 1). The hyperboloid model was used for all vines except those trained using the Head system, for which the volume of a cylinder (*Volume* = *π r*^*2*^* l*) of length (*l*) and radius (*r* = average of *r*_*m*_ + *r*_*b*_) was used. Cordon volume for Head-trained vines was approximated using the formula for a segmented cylinder:

$${Volume}_{Seg.Cyl.}=\frac{h}{2}\pi *\left({\left(\frac{{r}_{m}}{2}\right)}^{2}+ {\left(\frac{{r}_{b}}{2}\right)}^{2}\right)$$

Cordons and sub-cordons for all other vines were estimated using the formula for a regular cylinder.

Vine wood density was calculated experimentally in the laboratory by oven-drying sections of the vine, weighing them and then measuring the volume of water displaced [19]. We estimated wood density for the stump, trunk, cordon and sub-cordons of different age vines using Chardonnay (24 and 33 years old in 2018) for vines older than 20 years, and Cabernet Sauvignon (17 years old in 2018) for younger vines 20 years of age or younger.

For each block, we estimated vine wood volume for 3 to 10 vines chosen randomly, but selected at spatial intervals to reflect prominent variations in slope or aspect that might define a site. The larger and more topographically variable a block, the more vines were measured. Only live, healthy vines were included. Carbon was calculated for each vineyard block by averaging the volume of woody biomass in the sampled grapevines and multiplying the per-vine average by vine wood density. Vineyard managers provided data on varietal, age and planting density (number of vines per block), as well as the acreage of each block. This information was used to extrapolate C stored across each block, as well as to inform the sample size per block. Planting density averaged 1866 vines/ha (range: 1196–4480 vines/ha).

#### Belowground

For this reservoir, we estimated the contribution of wood in the stump and proximal root ball (but not fine roots) using allometric relationships derived experimentally from laboratory measurements of excavated vines [19]. In samples collected from old vines (age > 20 years), average stump weight was estimated at 51.8% of stem weight, while stump weight for younger vines (age ≤ 20 years) was estimated at 69.8% of stem weight.

To estimate C as a fraction of woody biomass, we multiplied biomass by 0.5 (i.e., carbon is approximately 50% of woody biomass by weight) based on the published average C content of vine wood [23, 25]. To estimate woody C per unit area, we calculated the average mass of the vines sampled, multiplied by the number of vines in that block and divided by the area (in hectares) of that block.

To estimate the annual C increment due to growth, we returned twelve-months after our initial sampling to resample the same vines—repeating our measurements of woody biomass. The C samples measured initially (Year-0) were subtracted from the samples measured one year later (Year-1) to give the delta, or 1-year C increment.

We measured soil organic carbon (SOC) for the top 30 cm of soil. The soils of the study area are primarily well-drained loams that vary in slope from 0 to 30%. We used USDA soil survey maps (https://websoilsurvey.nrcs.usda.gov/app) to identify prominent variation in soil types across the study area and to ensure that our sampling effort captured major differences in soil organic matter present across ranches and vineyard blocks. Sampling locations were recorded with a handheld global positioning system (GPS) unit.

To collect soil samples, vegetation and roots were removed from a roughly 0.7 × 0.7 m square area. From the center of this area, we used a 2.4 cm diameter hand auger to extract a column (soil core) from the top 30 cm of soil. Soil from the core was thoroughly mixed (homogenized), dried and sieved through a 2 mm filter. The < 2 mm soil fraction was ground to pass through a 0.25 mm sieve and then used to fill capsules for combustion analysis. All samples were analyzed at the Stable Isotope Facility at the University of California, Davis using a PDZ Europa ANCA-GSL elemental analyzer interfaced to a PDZ Europa 20–20 isotope ratio mass spectrometer (Sercon Ltd., Cheshire, UK). Additionally, we used a bulk density ring (height = 6.0 cm; diameter = 8.25 cm) to collect soil samples used to measure soil bulk density. For this analysis, we weighed clods dipped in impermeable paraffin and measured their volume by water displacement [26].

To estimate the annual C increment due to growth and incorporation of organic matter into soil, we returned twelve-months after our initial sampling to resample the same sites—repeating our measurements of woody biomass and collecting additional soil samples in sites adjacent to the initial samples. The C samples measured at Year-0 were subtracted from the samples measured at Year-1 to give the delta, or 1-year C increment. Year-1 soil samples were approximations of Year-0 cores, taken within ~ 3 m of the original core sites as a result of the accuracy limitations of the GPS used.

### Wildland carbon & increment

To estimate woody C in wildlands, we developed a sampling protocol stratified by vegetation type and coverage area, such that the number of sample plots per vegetation type was a function of the relative area of each type across the total wildland acreage. Sampling plot design followed USDA protocol [27] and consisted of a circular plot with 15 m diameter placed in representative sections of natural vegetation, such that extremely dense or bare patches, as well as edges, were avoided. Measurement of vegetation woody biomass within each plot was based on Brown et al. [28] and subsequent updates), in which the diameter at breast height (DBH; measured at 1.3 m above highest point on ground) was recorded for all individual trees with DBH > 5.0 cm. For shrubs, all woody shrub crown diameters were measured along two orthogonal axes and used as input for allometric equations for the closest corresponding species. For dead and downed stems, we measured base diameter and length for all those with a base diameter > 10.0 cm. All standing live trees and shrubs, dead standing and dead downed wood meeting the threshold size criteria were included in the sample with their dimensions recorded and identified to species level where possible. A metal tag with a unique identifying number was nailed to each measured stem.

Carbon in wildlands was measured for aboveground woody biomass only. For aboveground biomass, we used existing published allometric equations for trees and shrubs (e.g., [29, 30]). In the case of species with no published allometric equation to estimate volume, generic shrub or tree equations available through the USDA Forest Service were used [31]. Additionally, some aboveground remnant woody biomass was estimated using equations from Smith et al. [25]. Belowground woody C was not estimated in wildlands.

To estimate woody C increment in wildlands and in order to compare 2009 C stocks with 2018 stocks as precisely as possible, we measured aboveground C in the same 2009-established plots (identified by geographic coordinates recorded on a handheld GPS) using the same methodology used in 2009 [18]. The Williams et al. [18] method measures woody biomass in three adjoining 10 × 10 m subplots (total area is 10 × 30 m = 300 m2), where the species identity and diameter at breast height (DBH = 1.3 m above ground) are recorded for all stems > 5 cm DBH. After measuring all qualifying stems in the plot boundaries using this methodology, we used species- and genus-specific allometric equations (see Williams et al. [18] for sources used) to estimate total woody biomass and subsequently carbon mass [equal to 50% of woody biomass) for all stems. The per-stem C mass was summed for each subplot and plot and we compared these values to those from 2009 to estimate the incremental C accumulation that occurred in the intervening period.

### Statistical analysis

All statistical analyses were conducted in R 3.4.4 [32].

## Results

Total C stored in perennial vine woody biomass averaged 6.5 Mg/ha (2018 data) across all properties (vineyard blocks). Carbon stocks in adjacent uncultivated wildland woody biomass varied from 48.4 to 120.2 Mg/ha across the 5 vegetation types sampled (Table 1) and increased by 11.1% (std. dev. 52.4%) in oak dominated habitats between 2009 and 2018, demonstrating the significant carbon storage value of natural habitat conservation.

**Table 1:** Wildland average carbon storage by vegetation type

Vegetation Type	n	Ave. C (Mg/Ha)	Dominant Genera (by mass)
Russian River Riparian	6	48.4	*Populus, Sambucus, Juglans, Acer*
Oak Woodland	5	71.0	*Quercus*
Creek Riparian	3	73.6	*Populus, Quercus, Juglans, Fraxinus*
Oak Savannah	5	114.0	*Quercus*
Oak Madrone Woodland	3	120.2	*Quercus, Arbutus*

Average carbon (Mg/Ha) for 22 wildland vegetation plots sampled following Pearson et al. (27) and stratified a priori into 5 vegetation types based on dominant vegetation and topographic position

For the individual vines measured, the average estimated annual increase in woody C content was 38% over the course of the year considered. We found vine age to be a significant predictor (*p* <  < 0.001) of annual C increment in woody biomass across the vineyard blocks and varietals we measured. There was considerable variation in the per-vine increment, as indicated by the high standard deviation (57%). Much of the variability in annual increment came from differences in vine age: on average, young vines, which tended to be both small and fast-growing, added a larger percentage of biomass each year, and had higher variability in growth rates than older vines. For example, vines aged 2–5 years had standard deviation values more than three times any other 5-year grouping.

**Fig. 2:**
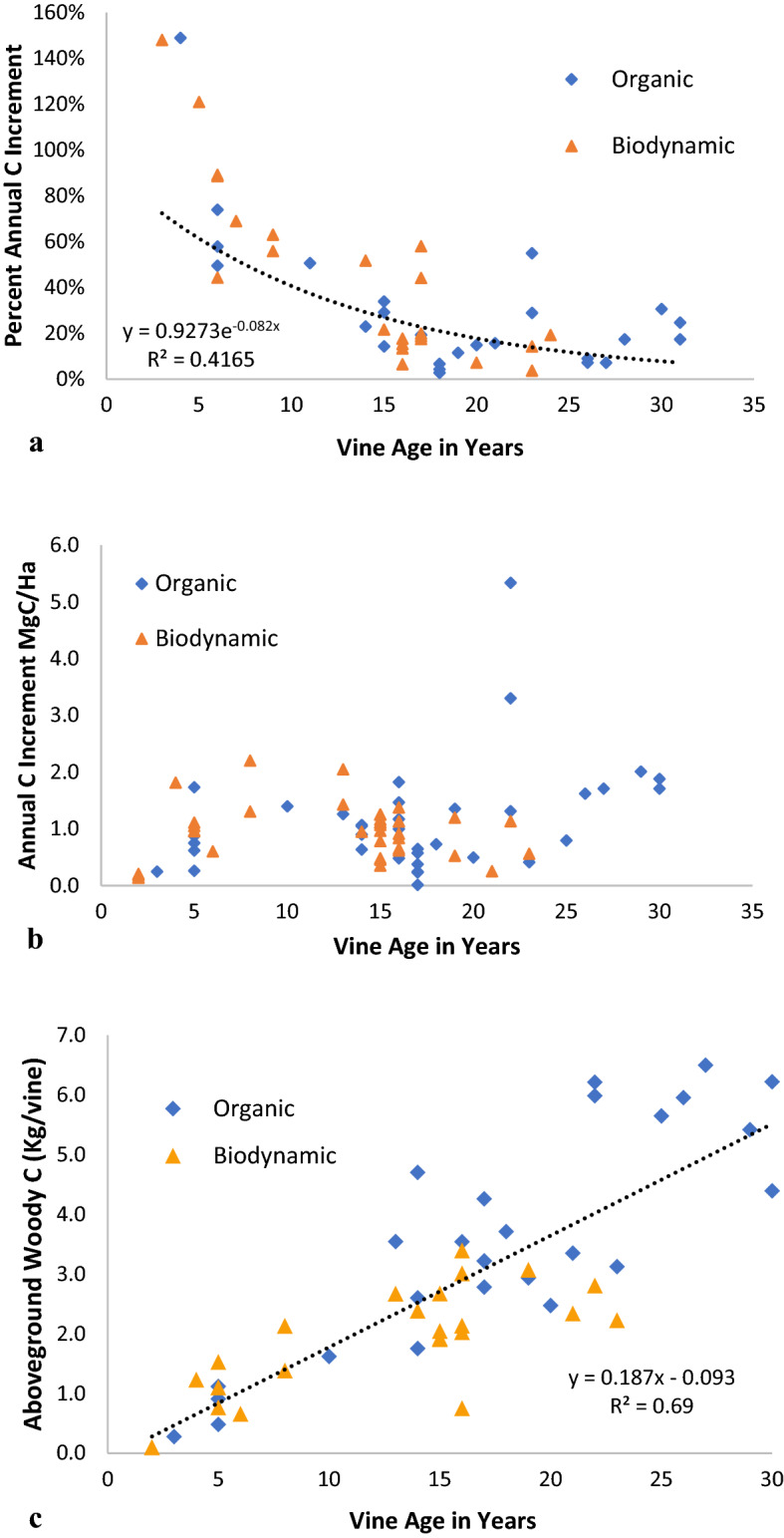
Annual C increment in vine woody biomass relative to vine age and management type. **a** shows the C increment as a percentage of grape vine woody biomass fitted with a negative natural logarithm trendline. **b** shows the increment in terms of additional C per hectare (Ha) added to the landscape. **c** shows cumulative C in woody biomass per vine by age

**Fig. 3:**
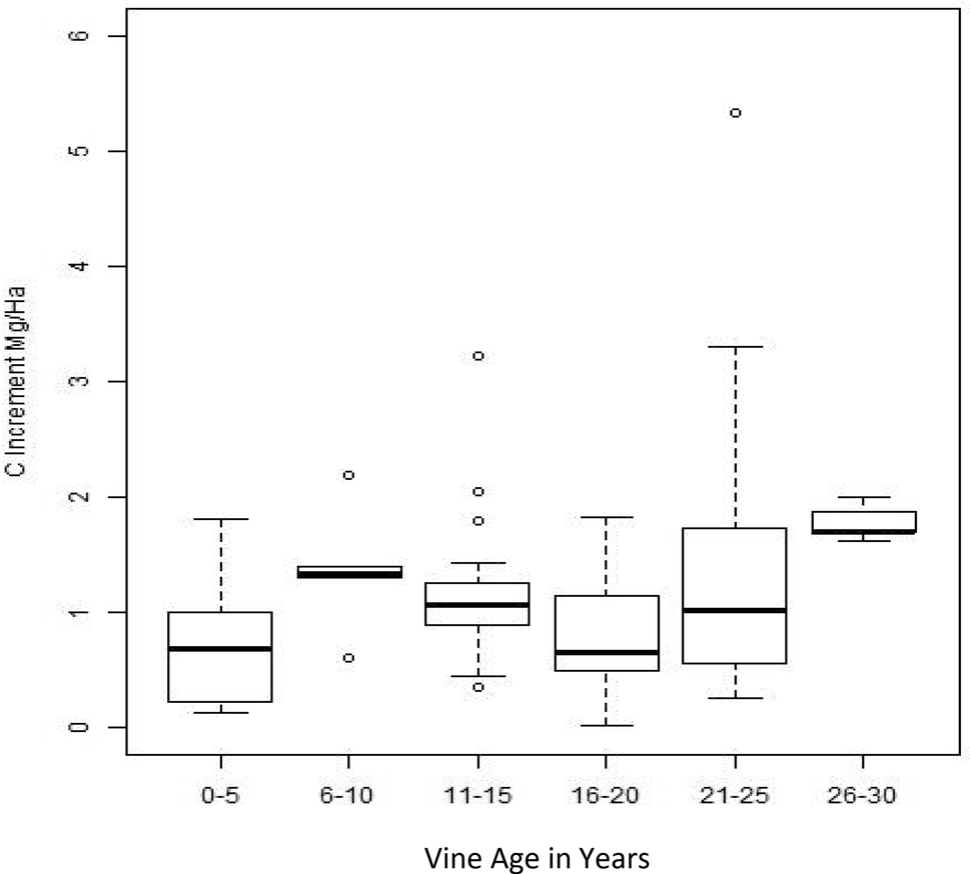
Landscape C increments of vine woody biomass by 5-year age class. This boxplot shows median values (dark lines) and quartile ranges of landscape C increments (Mg/Ha) from woody biomass for vines by 5-year age class (x axis)

Although young vines grew faster than older vines and added a greater percentage of woody biomass each year, older vines had more overall woody biomass and, as a result, the net C additions were often equal to or greater than young vines (Fig. 2); there was a net increase in aboveground woody biomass in vines over time that amounted to 18.7% per year on average (Fig. 2c; R^2^ = 0.64). We found that on an annual basis different aged vines stored similar amounts of C and showed no clear trend with respect to age and the total amount of C added (Fig. 3). In absolute terms (i.e., not by increment), older vines predictably stored more woody biomass than younger vines (Fig. 2c). As an example, while vines 2–5 years old increased C in woody biomass by 126% on average over the year in question, they stored only between 0.2 and 2 Mg/Ha of C, whereas vines 20 years or older had an average annual growth increment of 15.4%, but stored a total of 8.3 Mg/Ha of C in aboveground woody biomass on average.

**Fig. 4:**
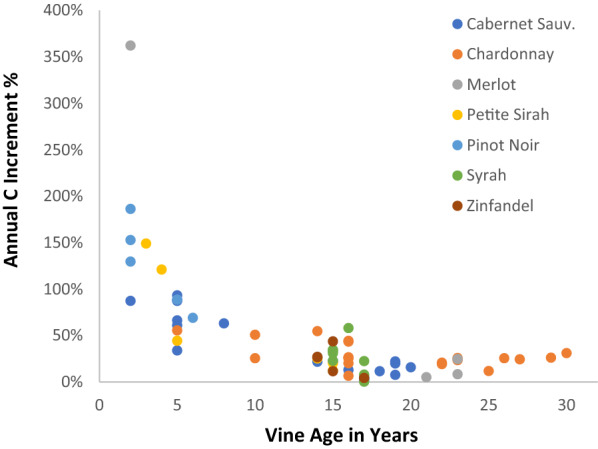
Annual C increment in woody biomass of grape vines by varietal. Increment is per block as a percentage of vine biomass. Slight negative values are likely a result of differences in water content of wood at the time of measurement

A summary of annual carbon increment in woody biomass of winegrape vines by site, management type, varietal and vine age is given in Table 2. Our a priori expectations of no significant effect of winegrape varietal on C increment were confirmed for the sixteen varietals considered (Fig. 4). A two-way ANOVA and Tukey’s Honestly Significant Difference test did show a single pair-wise significant difference (p < 0.004) between Cabernet Sauvignon (mean annual C increment = 26%) and Viognier (mean annual C increment = 61%). Viognier appeared to be the only varietal with appreciably above-average C increments, with nine of the top ten comparisons with the lowest p-values being between Viognier and another varietal.

**Table 2:** Summary of annual carbon (C) increment in woody biomass of winegrape vines by site, management type, varietal and vine age as measured in the years 2017 and 2018

Mgmt-Site	Varietal	Vine Age (2017)	2018 KgC/vine	Percent C Increment	Std Dev
O-1	CS	17	4.26	2.4%	3.0%
O-1	PS	17	3.22	− 3.4%	NA
O-1	Sy	17	2.78	10.1%	11.2%
O-1	Zi	17	3.22	4.3%	NA
O-2	Ch	25	5.65	11.7%	NA
O-2	Ch	26	5.95	25.4%	NA
O-2	Ch	27	6.50	24.3%	NA
O-3	CS	18	3.71	11.5%	NA
O-3	CS	20	2.47	15.7%	NA
O-3	CS	21	3.35	− 2.8%	NA
O-4	Ch	22	5.98	20.7%	NA
O-4	Ch	30	6.22	30.9%	NA
O-4	SB	13	3.54	23.0%	NA
O-4	Vi	30	4.39	29.5%	NA
O-5	Ch	16	3.54	17.2%	11.7%
O-6	CS	5	0.91	74.6%	19.3%
O-6	Me	23	3.12	− 1.8%	14.3%
O-6	PS	3	0.28	149.0%	NA
O-7	CS	19	2.93	6.8%	21.6%
O-8	Ch	10	1.62	50.7%	NA
O-8	Ch	29	5.42	26.1%	NA
O-9	CS	5	1.12	50.0%	22.8%
O-9	Ch	5	0.48	55.4%	NA
O-9	PS	14	1.75	25.3%	NA
O-9	Sa	14	4.70	17.0%	NA
O-9	Vi	22	6.21	81.4%	13.4%
O-9	Zi	14	2.60	26.7%	0.3%
B-1	CS	8	2.13	63.0%	NA
B-1	Ch	16	2.02	43.7%	0.6%
B-1	Mu	8	1.38	55.9%	NA
B-1	Ro	16	3.01	17.5%	NA
B-1	SB	13	2.67	51.0%	5.6%
B-2	CS	16	2.14	12.8%	NA
B-2	Gr	16	3.39	18.7%	NA
B-2	PS	5	1.53	44.3%	NA
B-2	PS	15	2.67	16.2%	5.7%
B-2	PV	15	1.91	17.7%	10.8%
B-2	PN	2	0.10	156.2%	28.5%
B-2	PN	6	0.66	69.0%	NA
B-2	Sy	15	2.05	31.0%	4.9%
B-2	Sy	16	0.75	58.0%	NA
B-2	Zi	15	1.92	27.6%	22.7%
B-3	CS	5	0.77	90.1%	4.2%
B-3	CS	14	2.38	21.6%	NA
B-3	CS	19	3.07	7.3%	12.7%
B-3	Ch	22	2.81	8.1%	15.7%
B-3	Me	2	0.10	362.1%	NA
B-3	Me	21	2.34	5.1%	NA
B-3	Me	23	2.23	24.6%	NA
B-3	PS	4	1.23	120.9%	NA
B-3	PN	5	1.10	88.5%	NA

Standard deviations are given where there were multiple blocks on a site planted with the same aged varietal. O, organic; B, biodynamic; CS, Cabernet Sauvignon; Ch, Chardonnay; Gr, Grenache; Me, Merlot; Mu, Muscat; PS, Petite Sirah; PV, Petit Verdot; PN, Pinot Noir; Ro, Rousanne; Sa, Sangiovese; SB, Sauvignon Blanc; Sy, Syrah; Vi, Viognier; Zi, Zinfandel

An evaluation of the effect of site, specifically the ranch property that a given vineyard block belonged to, was inconclusive. Although two-way ANOVA indicated “site” had a significant effect on increment (*p* = 0.03), further analysis with Tukey HSD multiple comparisons showed no significant differences between paired sites at the *p* = 0.05 level.

The increment analysis of soil organic carbon was inconclusive due to high variability among samples. For purposes of future comparison, we report only the baseline average SOC for ten sampled pits extrapolated to the per-hectare scale, which was 50.4 Mg/ha in the top 30 cm with a standard deviation of 9.4 Mg/ha.

## Discussion

Our results indicate there is a positive role that retention of older vines and native vegetation can play in on-farm (vineyard) carbon storage. Carbon storage in 5 native vegetation types averaged 81.7 Mg/Ha, more than an order of magnitude greater than the capacity of vines. Over a 9-year increment, C storage increased by 11.1% in oak dominated vegetation. Conservation of native perennial vegetation in buffers amidst vineyard blocks or adjacent to wetlands offers significant C accumulation benefits in addition to ecosystem services such as biodiversity, water quality, and erosion control.

Essentially, we combined a one-year increment analysis applied across different-aged vineyard blocks to produce a chronosequence of carbon storage rates over what is roughly the productive lifespan of most winegrape vines (aged 2–30 years), collectively represented as the curve in Fig. 2a. This chronosequence serves as a surrogate for direct, multiyear growth estimates. Longer-term studies that measure these rates directly would be able to estimate interannual variation in growth rates, as well as differences due to geography, varietal, and management actions (e.g., irrigation regime, cover crop selection) that we could not detect.

Despite the limitations of the chronosequence approach and the absence of multiyear rate data, we nevertheless found vine age to be an excellent predictor of annual C increment in woody biomass across the vineyard blocks and varietals measured, especially after the first six years when the annual variation in growth was highest. Our data show that despite the decrease in the annual rate of C accumulation, there is a net increase in aboveground woody biomass in vines over time that amounts to 18.5% per year on average.

Our research shows clearly that carbon in the woody biomass of vineyard landscapes increases over time—a pattern obviously not observed in annual crops. Given that perennial crops such as grape vines, with their relative longevity on the landscape and historical importance, have provided and continue to provide a range of ecosystem services at multiple spatial scales [13, 22, 33], the quantification of the carbon storage benefits of these cropping systems may play a role in land-use decision-making with regards to the viability of these systems relative to annual crops. This is especially expected in the face of climate change [34] and/or as part of a climate adaptation strategy [35].

The flattening of the curve in the annual decrease in carbon accumulation in vines roughly between 15 and 30 years of age (Fig. 2a) has positive management implications with respect to carbon storage. That is, if the quality and quantity of winegrapes produced by vines ≥ 15 years old are acceptable—and frequently they are, as evidenced by wines that put “old vine” on the label—then these older vines only improve with each additional year relative to vines younger than 15-years old. Given that it is not uncommon for vineyards to have productive vines older than 30 years, we note the need for empirical evidence to support that the plateau in C accumulation rate continues beyond this age.

Finally, while our analysis of soil organic C was inconclusive, this is not surprising given the short time period (one year) considered. Based on what other researchers have found (e.g., [36, 37], we expect that longer-term (e.g., ≥ 5 years) evaluations would potentially show both a clearer pattern of C accumulation/loss and variation due to differences in management approach, such as cover cropping, mulching and tillage [36–38]. There are also more sensitive ways to estimate soil carbon that we did not employ, such as the net ecosystem CO2 exchange approach [39] that might be recommended. Either way, soil C is potentially the most significant source of C in a vineyard ecosystem [18] and future research that examines how that reservoir changes over time and in response to different management practices will play a vital role in a developing a comprehensive understanding of C dynamics in this important agroecosystem.

## Conclusion

In conclusion, the findings presented here offer the first published estimates of C accumulation rates in vineyard ecosystems. Other studies have quantified C in vineyard systems, surrounding wildlands, soils, and in the vines themselves [8, 18, 19, 40–42], but none that we know of has estimated C accumulation rates or the effect of age and varietal on those rates. Furthermore, this study is the first to apply age-specific wood density measurements for estimating vine biomass and C content. While further improvements in C measurement precision are both possible and needed (e.g., volumetric precision, quantifying differences between the range of varietals, greater resolution of rates for vines of different ages), this study nevertheless advances the state of our understanding with regards to quantifying C storage in both vineyard systems in particular, and in perennial woody crops generally, where there is similar potential to provide ecosystem services including carbon storage while generating agricultural productivity [43].

Evidence from this study suggests that organic viticulture has the potential to generate a range of ecosystem services at multiple scales including mitigation of greenhouse gas emissions through long-term carbon storage. Advancing research in newly emerging areas, such as regenerative practices, would benefit from establishing baseline assessments of ecosystem services both within and adjacent to vineyards. This is especially true for quantifying C, but also for other ecosystem services such as nutrient cycling and biodiversity. As Biasi et al. [44] conclude, most older vineyards throughout the old and new world Mediterranean biome are placed within a mosaic of production and natural habitats, which require more synoptic approaches to ecosystem service benefits. Other recent studies [45–48] point to an emerging consensus on the role that vineyard block configuration, habitat remnants within vineyards and native genetic resources play in boosting overall biodiversity value [44, 49]. Our hope is that future studies will integrate these approaches to further demonstrate qualitatively and quantitatively how vineyards and various vineyard management systems, such as native perennial vegetation conservation, can contribute to ecosystem service provision and other positive environmental benefits [13, 22, 50, 51].

## Data Availability

Fetzer Vineyards retains proprietary rights over the datasets supporting the conclusions of this article, but is willing to make them available for non-commercial purposes upon request.

## Abbreviations

C: Carbon
GIS: Geographic Information System
USDA: United States Department of Agriculture
